# 1860. Long COVID Incidence by Sociodemographic Factors in a Large National Payor Database

**DOI:** 10.1093/ofid/ofad500.1688

**Published:** 2023-11-27

**Authors:** K J Craig, Amanda Zaleski, Michael Wiggins, Leah Altieri-Alger, Dorothea Verbrugge, Richard R Watkins

**Affiliations:** fda, fdaf, Alaska; CVS, Aurora, Ohio; CVS, Aurora, Ohio; CVS, Aurora, Ohio; CVS, Aurora, Ohio; CVS, Aurora, Ohio

## Abstract

**Background:**

Race/ethnicity (R/E) may be an independent risk factor for developing long COVID or post-acute sequelae SARS-CoV-2 infection (PASC). Evaluation of real-world data will likely reveal PASC-related sociodemographic (e.g., R/E and insurance type) disparities.

**Methods:**

A retrospective claims cohort analysis of Medicare, Medicaid, and Commercial fully-insured members (N=8,447,882) between October 2021 to December 2022 was conducted to identify members with PASC (ICD-10, U09.9). Overall differences in PASC estimated incidence and symptomatology and its duration were explored with post-hoc analyses to examine the influence of R/E and insurance type.

**Results:**

Overall PASC estimated incidence (n = 51,649) was 0.61% and was highest among American Indian/Alaskan Native (AI/AN, 0.91%) members, followed by White (0.78%), Black or Hispanic (0.49%), Pacific Islander (PI, 0.41%), and Asian or Unknown (0.34%) R/E groups. Compared to White members, all other R/E groups except AI/AN (*P* = 1.0) had lower incidence (all *P* < .01). Post-hoc analyses by R/E and insurance type indicated this trend persisted for Asian or Unknown members across all insurance types (all P < .001). Furthermore, compared to White members, lower PASC incidence (*P* < .001) was observed for a) Black Medicaid and Medicare; b) Hispanic Medicaid; and c) AI/AN Medicare members. The most frequent PASC symptoms identified by claims were pulmonary (36%), neurologic (17%), and cardiac (15%). Compared to White members (all *P* < .001), Black members had more cardiac symptoms in Medicaid and Medicare groups and more pulmonary symptoms in Commercially insured; and Hispanic or Unknown members had fewer pulmonary, neurologic, and cardiac symptoms among all insured. There were no differences in PASC symptom duration by R/E within insurance type (all *P* > .053).

**Conclusion:**

Hispanic and Unknown members had fewer claims for PASC than White members, which may indicate a lower disease burden or disparities in care access. Despite having overall less PASC estimated incidence than White members, Black members had more cardiac and pulmonary symptoms. Pulmonary symptoms were most frequently identified in all R/E groups. There was no association of PASC symptom duration between R/E within insurance type.

**Disclosures:**

**KJ Craig, PhD**, cvs Health Inc: employment|cvs Health Inc: Stocks/Bonds **Amanda Zaleski, PhD**, CVS: Employee|CVS: Stocks/Bonds **Michael Wiggins, MPH**, CVS: Employee|CVS: Stocks/Bonds **Leah Altieri-Alger, MPH**, CVS: Employee|CVS: Stocks/Bonds **Dorothea Verbrugge, MD**, CVS: Employee|CVS: Stocks/Bonds **Richard R. Watkins, MD, MS, FACP, FIDSA, FISAC**, bioMerieux: Advisor/Consultant|CVS: Employee|CVS: Stocks/Bonds

